# Trends in NRMP Data from 2007–2014 for U.S. Seniors Matching into Emergency Medicine

**DOI:** 10.5811/westjem.2016.10.31237

**Published:** 2016-11-23

**Authors:** David E. Manthey, Nicholas D. Hartman, Aileen Newmyer, Jonah C. Gunalda, Brian C. Hiestand, Kim L. Askew, Cedric Lefebvre

**Affiliations:** * Wake Forest School of Medicine, Department of Emergency Medicine, Winston-Salem, North Carolina; † Henry Ford Hospital, Department of Emergency Medicine, Detroit, Michigan

## Abstract

**Introduction:**

Since 1978, the National Residency Matching Program (NRMP) has published data demonstrating characteristics of applicants who have matched into their preferred specialty in the NRMP main residency match. These data have been published approximately every two years. There is limited information about trends within these published data for students matching into emergency medicine (EM). Our objective was to investigate and describe trends in NRMP data to include the following: the ratio of applicants to available EM positions; United State Medical Licensing Examination (USMLE) Step 1 and Step 2 scores (compared to the national means); number of programs ranked; and Alpha Omega Alpha Honor Medical Society (AOA) membership among U.S. seniors matching into EM.

**Methods:**

This was a retrospective observational review of NRMP data published between 2007 and 2016. We analyzed the data using analysis of variance (ANOVA) or Kruskal-Wallis testing, and Fischer’s exact or chi-squared testing, as appropriate to determine statistical significance.

**Results:**

The ratio of applicants to available EM positions remained essentially stable from 2007 to 2014 but did increase slightly in 2016. We observed a net upward trend in overall Step 1 and Step 2 scores for EM applicants. However, this did not outpace the national trend increase in Step 1 and 2 scores overall. There was an increase in the mean number of programs ranked by EM applicants over the years studied from 7.8 (SD4.2) to 9.2 (SD5.0, p<0.001), driven predominantly by the cohort of U.S. students successful in the match. Among time intervals, there was a difference in the number of EM applicants with AOA membership (p=0.043) due to a drop in the number of AOA students in 2011. No sustained statistical trend in AOA membership was identified over the seven-year period studied.

**Conclusion:**

NRMP data demonstrate trends among EM applicants that are similar to national trends in other specialties for USMLE board scores, and a modest increase in number of programs ranked. AOA membership was largely stable. EM does not appear to have become more competitive relative to other specialties or previous years in these categories.

## INTRODUCTION

Since 1978, the National Residency Matching Program (NRMP) has published data demonstrating characteristics of applicants who have matched into their preferred specialty in the NRMP main residency match. Data available on the NRMP website approximately every two years include a summary entitled “Charting the Match Outcomes,” as well as the results of the NRMP Applicant Survey results and the most recent Main Residency Match data.^1^–^10^ Although this information is publically available and fairly easy to interpret, there is limited information about trends within these published data for students matching into emergency medicine (EM).

In a recent commentary, a respected EM educator stated in the American College of Emergency Physicians (ACEP) News, “It is getting tougher every year to match in EM. In 2015, the average United States Medical Licensing Exam (USMLE) Step 1 score of a student who matched in EM was 230, up from 219 in 2006.”^11^ This sentiment has been echoed in multiple arenas by EM residency leaders as well as by those tasked with advising medical students applying for residency positions, including medical student educators in EM. Therefore, we sought to evaluate the available data for any trends that might suggest that EM was becoming more competitive.

Although an increased average USMLE Step 1 or 2 score may indicate that the quality of applicants to EM has improved and therefore the competitiveness of the specialty has grown, it may also be that overall Step 1 or 2 scores are increasing across all medical students. Other ways to suggest increased competitiveness in EM applicants would include an increased number of applicants per available spot. As the Alpha Omega Alpha Medical Honor Society (AOA) is considered by many to be a marker of a more competitive applicant, an increasing percentage of applicants attaining AOA status would suggest a trend towards EM becoming a more competitive specialty in which to match. Our objective was to investigate and describe secular trends in the NRMP data to include the ratio of applicants to available EM positions, USMLE Step 1 and Step 2 scores (taken in context with trends in all match participant scores), number of programs ranked by each student, and AOA membership among U.S. seniors matching into EM.

## METHODS

We performed a retrospective cohort analysis of NRMP data generated between 2007 and 2016. Summary data were available for students participating in the match process in 2007, 2009, 2011 and 2014, with limited data available for 2016. Data included both successful and unsuccessful participants, as well as both U.S. and international medical graduates (categorized as “independent” in the NRMP products).

USMLE score distributions for students matching in EM were available as proportions of participants scoring within 10-point intervals. As an example, in 2009 15.52% of U.S. participants successfully matching in EM had a Step 1 score between 201–210. To transform these categories into continuous data distributions, we calculated weighted averages using the midpoint of each range to generate an overall average score and variance. Other data elements were taken directly from the NRMP reports without transformation or alteration of definitions. We used analysis of variance (ANOVA) to compare continuous variables when sufficient detail was available from NRMP sources. The data were sufficient for EM applicants, but not for the overall cohort of students participating in the U.S. residency match. Therefore, comparison of means and reported standard deviations involving the total U.S. cohort were performed without hypothesis-based testing. Equality of variances assumption was violated for the evaluation of number of programs ranked per year, so we performed Kruskal-Wallis testing in lieu of one-way ANOVA, and used Dunn’s test for post-hoc comparisons. We calculated Fisher’s exact test or chi-squared test as appropriate to compare categorical data. All statistics were two-tailed, and a p<0.05 was held to represent statistical significance. Given that the sizes of the cohorts were fixed, we did not perform sample-size calculations when the intent was to use all available data. We calculated statistics using Stata IC 11.2 (College Station, TX). As no subject level data were provided, the Biomedical Institutional Review Board at Wake Forest University Health Sciences determined this study to be non-human subjects research and exempt from formal review.

## RESULTS

The total number of U.S. and independent applicants for EM increased steadily from 1,669 in 2007 to 2,476 in 2016 (Table 1), while the total number of EM positions available in the main match increased from 1,384 to 1,895 over the same time period. Across U.S. and independent seniors (matched and unmatched), the ratio of applicants to available EM positions remained relatively flat from 2007 to 2014 but did increase in 2016. The proportions of U.S., independent, matched, and unmatched students remained stable from 2007 to 2014 (χ^2^ (9) = 16.67, p=0.054).

We observed a statistically significant upward trend in overall USMLE Step 1 and Step 2 scores for EM applicants in the time period studied (Table 2). However, the mean USMLE Step 1 exam score for matched U.S. seniors in EM increased at a rate similar to all U.S. seniors. The mean USMLE Step 2 exam score for matched U.S. seniors in EM and other U.S. seniors rose by 16 and 17 points respectively from 2007 to 2014.

Table 3 shows the pattern of number of ranked programs for EM applicants. Overall, there was a statistically significant increase in the average number of programs ranked by EM applicants among the years studied (p<0.001 by Kruskal-Wallis testing). An overall decrease in the number of programs ranked by independent unmatched students from 2007 to 2014 (p=0.018 by Dunn’s test) was offset by the larger cohort of U.S. students who matched, which demonstrated a consistent year-to-year increase in the number of programs ranked from 2007 to 2014 (p<0.001 by Dunn’s test).

Across the study period, there was a statistical difference in the number of applicants who matched in EM and were AOA (p=0.043 by Fisher’s exact test), primarily due to a drop in the number of AOA students in 2011 (Table 4). There was no statistical difference in AOA membership among students who did not match (p=0.30 by Fisher’s exact test).

## DISCUSSION

Although the data reviewed here regarding the EM residency match are publicly available, we attempted to consolidate the information and interpret trends. The growth of EM as a specialty has coincided with a perception that entry into the field has become more competitive in recent years. The findings in our study challenge this assertion in a few ways while suggesting a possible source for this perception. First, while the number of EM applicants has steadily increased each year, this increase has mostly been matched by an increase in the number of EM positions available in the match. The 2016 match revealed the first increase during this time period in the ratio of applicants to positions available. Time will tell whether this is an anomaly or the beginning of a trend. Second, while scores on entry examinations have increased in EM applicants, this mirrors the increase in USMLE scores seen in students applying to all specialties. Third, there appears to be a small but statistically significant increase in the number of programs ranked by individual applicants, driven largely by an increase in programs ranked by students who ultimately match. Fourth, after accounting for minor year-to-year variation, the percentage of EM applicants who are members of AOA has also remained fairly constant. Thus, by most of these measures, entry into EM has not become more competitive over the past decade, with the possible exception of the 2016 match, for which complete data are not yet available. The effect of the increased number of programs ranked by ultimately matched applicants is not clear, but may be driving a perception of competitiveness by lessening opportunities for students whose applications are less competitive.

Despite the lack of relative change in the composition of the applicant pool with regard to test scores and AOA status, there are other domains in which applicants to EM may be becoming increasingly competitive. For example, in this analysis we are not able to comment on changes in clerkship or medical school grades or interview performance, factors that programs and applicants both rate as important.^12^ These factors may predict success as well as the factors that have been quantified and examined here.^13^ Test scores and memberships in an honorary society should not be taken as evidence of an entirely unchanging applicant pool. However, in light of previously reported difficulties that EM faculty members have in accurately assessing applicants for letters of evaluation and in predicting position on rank lists, it is important that the relative meaning of these scores and designations be understood.^14^, ^15^

Another area of recent discussion has involved applicant behavior regarding rank lists and interviews. We used programs ranked as a proxy for interviews taken, since the data for this metric were more complete and could be better analyzed. There does appear to be a small but apparent increase in mean programs ranked by successful U.S. applicants. The decrease in the number of ranked programs seen in the unmatched applicant cohort may reflect the reality of fewer interview opportunities for those applicants at the lower end of the competitive spectrum. We also noted that independent applicants, both matched and unmatched, appeared to rank fewer programs than their U.S. counterparts. This is likely due to independent applicants being granted fewer interviews.

Appropriately assessing growth in both medical school enrollment and available residency positions necessitates close monitoring of the applicant pool. Our findings suggest that over the past decade these trends have been appropriately matched and the quality of the applicant pool for EM has remained relatively stable. Further study is needed to more accurately identify changes in interviewing behavior among programs and applicants; there are trends suggesting more interviews are taken by competitive (and ultimately matched) applicants while less competitive applicants interview at and rank fewer programs. However, these trends bear further study before firm conclusions can be made.

## LIMITATIONS

This study relied upon data available on the NRMP website. We requested additional data to allow more in-depth analysis; however, these data were not accessible to the authors. Information published on the EM match in 2016 is limited at this time, thus not allowing the authors to make further analysis from the two most recent match years. As more data are published by the NRMP, these analyses should be revisited. Additional data on residency applicants to EM, such as characteristics on a standardized letter of evaluation (SLOE), are not accessible to allow for more granular analysis. Finally, the study looks at the general pool of applicants, and not at specific cohorts or individual applicants. Intangible, and therefore unquantifiable, characteristics of the applicant may have as much impact on the competitiveness of the application as numeric data.

## CONCLUSION

NRMP data demonstrate trends among EM applicants that are similar to national trends in other specialties for USMLE board scores, and stability in number of programs ranked and AOA membership. EM does not appear to have become more competitive relative to other specialties or previous years in these categories.

## Figures and Tables

**Table 1:** Number of applicants to emergency medicine residency programs from 2007 to 2016. Data are presented as counts and percentages. Subgroup data not available (n/a) for 2016.

	2007	2009	2011	2014	2016
Total number of applicants	1669	1817	2025	2106	2476
Total number of positions	1384	1515	1626	1786	1895
Applicant to position ratio	1.21	1.20	1.25	1.18	1.30
U.S. matched applicants	1092 (65.4)	1153 (63.5)	1259 (62.2)	1371 (65.1)	n/a
U.S. unmatched applicants	89 (5.3)	92 (5.1)	137 (6.8)	106 (5.0)	n/a
Independent^*^ matched applicants	265 (15.9)	317 (17.5)	330 (16.3)	370 (17.6)	n/a
Independent unmatched applicants	223 (13.4)	255 (14.0)	299 (14.8)	259 (12.3)	n/a

*Independent,* international applicant.

**Table 2:** USMLE scores among all U.S. seniors participating in the match and U.S. seniors who matched in EM. Scores are presented as mean (standard deviation). P values were generated via ANOVA.

	2007	2009	2011	2014	P value F statistic
U.S. EM step 1	220 (18.5)	222 (17.8)	221 (17.5)	230 (16.9)	<0.001 F_3,4737_ = 83.09
All U.S. step 1	220 (20.3)	224 (20.3)	225 (20.6)	230 (18.8)	n/a
U.S. EM step 2	227 (19.5)	229 (19.2)	234 (17.9)	243 (14.9)	<0.001 F_3,3899_=156.10
All U.S. step 2	225 (22.3)	230 (21.8)	234 (20.4)	242 (16.6)	n/a

**Table 3:** Trends in numbers of programs ranked. Data are presented as means (standard deviation).

	2007	2009	2011	2014	P value χ^2^
All EM applicants	7.8 (4.2)	8.0 (4.3)	8.5 (4.6)	9.2 (5.0)	<0.001 χ^2^(3)=127.5
U.S. matched	9.5 (3.4)	9.8 (3.4)	10.7 (3.4)	11.6 (3.4)	<0.001 χ^2^(3)=293
U.S. unmatched	5.2 (3.4)	5.3 (3.6)	4.9 (3.5)	4.4 (2.5)	0.11 χ^2^(3)=6.1
Independent matched	5.9 (3.9)	6.3 (4.0)	6.7 (4.0)	6.7 (4.4)	0.13 χ^2^(3)=5.7
Independent unmatched	2.8 (2.8)	3.0 (3.1)	3.1 (3.1)	2.5 (2.9)	0.02 χ^2^(3)=9.5

*Independent*, international applicant.

**Table 4:** AOA medical society status among U.S. seniors pursuing a match in emergency medicine.

	2007	2009	2011	2014
Percent AOA among matched U.S. seniors	12.4	10.9	9.1	12
Percent AOA among unmatched U.S. seniors	1.1	3.3	0.7	4.1

*AOA,* Alpha Omega Alpha Medical Honor Society.

## References

[1] (2016). National Resident Matching Program, Results and Data: 2016 Main Residency Match®.

[2] National Resident Matching Program, Data Release and Research Committee (2015). Results of the 2015 NRMP Applicant Survey by Preferred Specialty and Applicant Type.

[3] National Resident Matching Program, Data Release and Research Committee (2013). Results of the 2013 NRMP Applicant Survey by Preferred Specialty and Applicant Type.

[4] National Resident Matching Program, Data Release and Research Committee (2011). Results of the 2011 NRMP Applicant Survey by Preferred Specialty and Applicant Type.

[5] National Resident Matching Program, Data Release and Research Committee (2009). Results of the 2009 NRMP Applicant Survey by Preferred Specialty and Applicant Type.

[6] National Resident Matching Program, Data Release and Research Committee (2008). Results of the 2008 NRMP Applicant Survey by Preferred Specialty and Applicant Type.

[7] (2014). National Resident Matching Program, Charting Outcomes in the Match, 2014.

[8] (2011). National Resident Matching Program, Charting Outcomes in the Match, 2011.

[9] (2009). National Resident Matching Program, Charting Outcomes in the Match, 2009.

[10] (2007). National Resident Matching Program, Charting Outcomes in the Match, 2007.

[11] House H (2016). The 2016 NRMP: Emergency medicine remains one of the most popular specialties. ACEP Now.

[12] McCann S, Nomura J, Terzian W (2016). Importance of the emergency medicine application components: the medical student perception. J Emerg Med.

[13] Bhat R, Takenaka K, Levine B (2015). Predictors of a top performer during emergency medicine residency. J Emerg Med.

[14] Grall K, Hiller K, Stoneking L (2014). Analysis of evaluative components on the standard letter of recommendation (SLOR) in emergency medicine. West J Emerg Med.

[15] Oyama L, Kwon M, Fernandez J (2010). Inaccuracy of the global assessment score in the emergency medicine standard letter of recommendation. Acad Emerg Med.

